# An Efficient End-to-End Multitask Network Architecture for Defect Inspection

**DOI:** 10.3390/s22249845

**Published:** 2022-12-14

**Authors:** Chunguang Zhang, Heqiu Yang, Jun Ma, Huayue Chen

**Affiliations:** 1 School of Automation and Electrical Engineering, Dalian Jiaotong University, Dalian 116028, China; 2 Traction Power State Key Laboratory, Southwest Jiaotong University, Chengdu 610031, China; 3 School of Computer Science, China West Normal University, Nanchong 637002, China

**Keywords:** surface defect detection, semantic segmentation, object detection, multi-task network

## Abstract

Recently, computer vision-based methods have been successfully applied in many industrial fields. Nevertheless, automated detection of steel surface defects remains a challenge due to the complexity of surface defects. To solve this problem, many models have been proposed, but these models are not good enough to detect all defects. After analyzing the previous research, we believe that the single-task network cannot fully meet the actual detection needs owing to its own characteristics. To address this problem, an end-to-end multi-task network has been proposed. It consists of one encoder and two decoders. The encoder is used for feature extraction, and the two decoders are used for object detection and semantic segmentation, respectively. In an effort to deal with the challenge of changing defect scales, we propose the Depthwise Separable Atrous Spatial Pyramid Pooling module. This module can obtain dense multi-scale features at a very low computational cost. After that, Residually Connected Depthwise Separable Atrous Convolutional Blocks are used to extract spatial information under low computation for better segmentation prediction. Furthermore, we investigate the impact of training strategies on network performance. The performance of the network can be optimized by adopting the strategy of training the segmentation task first and using the deep supervision training method. At length, the advantages of object detection and semantic segmentation are tactfully combined. Our model achieves mIOU 79.37% and mAP@0.5 78.38% on the NEU dataset. Comparative experiments demonstrate that this method has apparent advantages over other models. Meanwhile, the speed of detection amount to 85.6 FPS on a single GPU, which is acceptable in the practical detection process.

## 1. Introduction

The steel strip is one of the main products of the steel industry. As an important industrial material, the quality of steel directly affects the quality of the final products. Due to the limitations of production conditions, there are inevitably different types of defects on the surface of the steel strip, such as inclusion (In), patches (Pa), pitted surface (Pt), scratches (Sc), crazing (Cr), and rolled-in scale (Ri). With the development of industrial intelligence, efficient steel surface defect detection methods have become a recent research hotspot.

In practical terms, steel surface defect detection is currently facing three major challenges.

**Figure 1:**
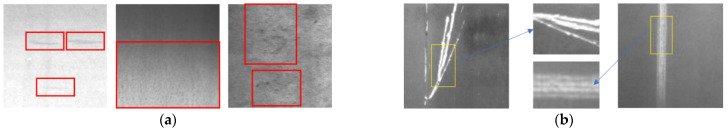
Challenges of defect inspection. (**a**) shows the low contrast between defects and background in the image. (**b**) shows the intra-class difference.

1. Low contrast, as shown in Figure 1a. Influenced by dust, metal surface reflection, etc., defects in the image have low contrast to the background.
2. Intra-class difference, as shown in Figure 1b. As a result of the inhomogeneity of the production process, the measurement, silhouette, and other characteristics of similar defects are quite different.
3. Small sample size. Because defects are not common in actual production, and fine annotation requires a lot of labor, data collection and annotation are very expensive in defect detection.

The above two reasons limit the size of the dataset, and the limited data brings difficulties to the training of the model.

With the development of artificial intelligence in recent years, there are three main research directions for surface defect detection.

1. Image-level defect classification: classify images according to defect types.
2. Object-level defect detection: identify each defect on the image and label its rough range.
3. Pixel-level defect segmentation: classify each pixel of the image and accurately segment defects.

Benefiting from the rapid development of deep learning, the task of image-level defect classification has been well resolved. The accuracy of defect classification has approached 100% [1,2,3]. However, this method is too crude. It can only classify images by a single defect type. In actual production, multiple defects may exist in the same image at the same time, which reduces the classification accuracy [4,5,6,7,8]. 

Object-level detection has a good performance on objects with clear and separable instances [9,10,11,12,13,14]. However, the performance is sharply reduced for those objects with unclear boundaries that are more like a texture and pattern.

Semantic segmentation performs well for targets based on fine-grained information and texture information [15,16,17,18]. However, it does not perform well on small objects with low contrast.

To overcome the above difficulties, an obvious approach is to combine object detection and semantic segmentation. However, using the two methods to process this task separately would be more computationally expensive [19,20,21,22]. Adopting a multi-task network not only saves computational costs but also has performance advantages because a multi-task network is equivalent to using more information to train the network [23,24,25,26,27]. The multi-task network learns more generalized features than the single-task network under the same dataset, which suppresses overfitting while optimizing the network feature extraction ability [3,28,29,30,31,32,33]. Therefore, we propose an end-to-end lightweight network for simultaneous defect segmentation and detection. 

Our model consists of one encoder and two decoders. The detection decoder is based on the state-of-the-art single-stage detection network yolov5 [32,33,34,35,36,37,38]. There are two main reasons:

1. The single-stage detection network is faster and easier to meet the real-time performance than the two-stage detection network;
2. Compared with the two-stage detection network, the single-stage detection network has better encoder optimization, which can give the segmented decoder better performance.

The segmentation decoder adopts a structure that fuses high-level and low-level feature maps. High-level feature maps extract richer multi-scale semantic information by using a Densely connected Depthwise Separable Atrous Spatial Pyramid Pooling module (DDWASPP) [39,40,41]. The low-level feature maps use Residually Connected Depthwise Separable Atrous Convolutional Blocks (resDWAB) to extract clearer spatial information. This architecture is chosen because this decoder architecture achieves the best balance of performance and speed on the defect detection task compared to other architectures (e.g., unet, hrnet, pspnet) [42,43,44,45,46]. We verify this in the experiments section. In addition, we also investigate the impact of the training strategy on the performance of our model. Experiments show that training segmentation first can effectively guide the detection task. It has better performance than end-to-end training and alternating training. Finally, our model achieves 79.37%mIOU, 78.97%mAP@0.5, and 85.6 FPS on a single GPU Tesla P100.

In summary, our main contributions are:

1. An efficient multi-task network is proposed, which combines the advantages of both methods while saving computational costs. It achieves the best overall performance. This proves that the method has certain generality and theoretical value.
2. A DDWASPP module is proposed, which is used to extract dense multi-scale features. Compared with other multi-scale feature extraction methods, this module has a significant computational advantage.
3. A resDWAB module is proposed, which is used to reinforce the spatial information of the encoder’s low-level feature maps, ensuring that it can provide useful information for the final prediction. Experiments show that this method can significantly improve segmentation performance with low computation.
4. A training strategy is investigated. Adopting the strategy of training the segmentation task first can improve the performance of the model, which is believed to provide a reference for other related research work.

## 2. Related Work

The methods based on computer vision for surface defect detection can be categorized into traditional detection approaches and deep-learning-based detection approaches [47,48,49,50,51]. Generally, traditional approaches include two parts: a feature extractor and a classifier. Whether the feature extractor can extract effective features directly determines the upper limit of the detection performance of the method [52,53,54,55,56,57]. Therefore, researchers of traditional methods pay more attention to the improvement of feature extractors. Nana et al. [58] used a modified local binary method for feature extraction of defect images. Although the above two methods can achieve good results, their robustness is poor, and it is difficult to distinguish multiple similar defects. Moe et al. [59] used a contrast adjustment thresholding method for the binarization of defect images. This method also has the problem of insufficient robustness. It has difficulty distinguishing defects with similar characteristics. Ni et al. [60] and Gan et al. [61] applied statistical-based defect detection algorithms to the field of rail detection and achieved good performance. However, this is mainly due to the advantages of smooth and flat rail surfaces, which provide a stable background and large differences from defects. The hot-rolled steel plate does not have such conditions at all.

In recent years, with the development of deep learning technology, deep learning-based machine vision algorithms have had great advantages over traditional algorithms. At present, many researchers have tried to apply deep learning to defect detection. Li et al. [62] fused the classification head on the basis of Hrnet, which increased the accuracy of defect classification with a small increase in the amount of calculation. Xu et al. [63] introduced conditional label vectors into GAN to generate more reliable adversarial examples and successfully achieved better results with a smaller sample size. However, the defect images used in the above three works are strictly screened; that is, each image contains only one type of defect, which is difficult to compare to the actual situation.

In order to solve the problem of irregular defect shape, He et al. [64] proposed a Multi-scale Feature Fusion Network (MFN) to improve the quality of Regions Of Interest (ROI) proposed by the Region Proposal Network (RPN). This successfully increased mAP by 10% over the baseline model. Hao et al. [35] proposed an improved backbone network using deformable convolution to enhance the feature extraction ability of the network. The model successfully improved the detection accuracy. Zhang et al. [65] used the improved YOLOV3 and the improved SSD for parallel prediction and fused the prediction results of the two to enhance the accuracy of prediction. Although this can improve the final detection accuracy, it consumes much more computing resources. Tao et al. [66] used a multi-attention mechanism to enhance the detection effect of the network on small objects. However, this method increases the difficulty of training and is difficult to apply to sophisticated scenes. However, the above two methods only achieved good results on the four defects of Pa, In, Sc, and Pt and did not solve the detection problem of Cr and Ri. Furthermore, the slow detection speed of the two-stage detection method means that it is difficult to apply practically. Dong et al. [67] used a Pyramid Feature Fusion Module and Global Context Attention Module to fuse multi-scale context information and added a boundary refinement block to improve defect segmentation. However, the detection speed is only 40 FPS. Zheng et al. [68] used the chained ASPP module to improve the deeplabv3+ network. The performance of model segmentation is enhanced, and the detection speed reached a practical level, but the detection accuracy is not high enough. In addition, the types of defects tested by the above two methods are too few; only three types, In, Pa, and Sc, so it has difficulty meeting the actual industrial needs. Zhang et al. [69] used an object detection algorithm and a semantic segmentation algorithm for the surface damage of the rails, respectively, and found that the two algorithms each have different advantages for different classes of defects.

Through the analysis of the above results, we believe that the six common surface defects of steel strips have different types of characteristics. In, Pa, and Sc have clear boundaries and strong instance features, which are very suitable for object detection algorithms. Cr and Ri are more like a textured pattern without sharp boundaries, which deteriorates the object detection performance. Pa and Pt have large scales and high contrast with the background, which are very suitable for semantic segmentation algorithms. In and Sc have high contrast with the background, but there are too many small complex structures, which makes accurate segmentation difficult. Although Cr and Ri are more like a textured pattern, they have little effect on the semantic segmentation algorithm. The only difficulty is that the contrast with the background is too low.

Based on the above analysis, this paper proposes a lightweight multi-task network, which combines the advantages of object detection and semantic segmentation, and can realize the detection and segmentation of six kinds of steel surface defects.

## 3. Methodology

**Figure 2:**
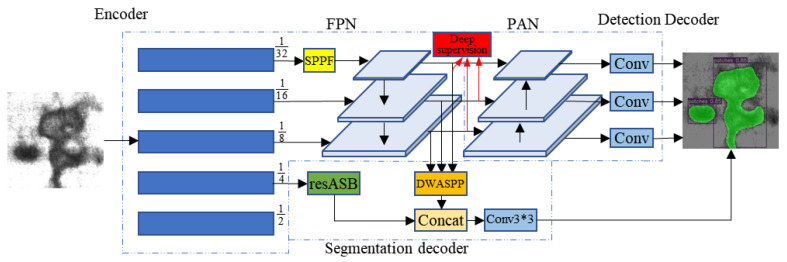
Architecture of the proposed model.

In this section, the structure and the training set of our model are fully illustrated. The structure of the network is illustrated in Figure 2.

Our model mainly consists of three parts: encoder, segmentation decoder, and detection decoder. The encoder uses CSPDarkNet53 as the backbone and FPN for semantic feature enhancement. The encoder is trained in a deeply supervised way during training. The detection decoder uses PAN on the basis of the backbone for spatial information enhancement. The enhanced information is then used to make predictions. The segmentation decoder uses the DWASPP module for semantic information enhancement and the resASB module for spatial information enhancement, respectively. After the two are fused, segmentation prediction is performed. Finally, the prediction result of the detection decoder is combined with the result of the segmentation decoder to obtain the final prediction result.

Because surface defect detection is a time-sensitive task, and compared with the two-level network, the single-stage network can better use the training advantages of the multi-task network, we chose the most advanced single-stage detection network, yolov5, as the backbone network.

### 3.1. Model Structure

First, input a raw image to the network, and extract the multi-level features using the encoder. Next, feed these multi-level feature maps to the semantic segmentation decoder and object detection decoder, respectively. The object detection decoder first adopts the PAN structure for multi-level features fusion. Then, three different resolution feature maps obtained by PAN feature fusion are used to predict large, medium, and small objects, respectively.

The semantic segmentation decoder first extracts dense multi-scale information on high-level feature maps using DDWASPP. Next, resDWAB are used to extract spatial features of low-level feature maps while preserving resolution. Finally, the high-level feature maps and low-level feature maps are fused for prediction.

### 3.2. Object Detection Decoder

The object detection decoder adopts an anchor-based multi-scale detection scheme. The decoder consists of two parts, PAN and detection head. The FPN [70] structure in the encoder transfers semantic information from top to bottom. The PAN [71] in the detection decoder transmits spatial information through a bottom-up path. Combining these two structures can obtain better feature maps. Next, the multi-scale feature maps obtained by PAN are directly used for prediction. Then, three prior boxes with different aspect ratios are assigned to each anchor point of the multi-scale feature maps during prediction, and the detection head predicts the position offset of the prior box, the scaling of height and width, the prediction confidence, and the corresponding probability of each category. Finally, the most suitable prediction box is selected from many candidate boxes by means of non-maximum suppression.

### 3.3. Semantic Segmentation Decoder

At present, there are three main structures for semantic segmentation, U-shape structure (Unet), high-resolution parallel structure (Hrnet), and Spp structure (PSPnet). The above three structures have their own advantages. The U-shape structure can obtain better-detailed information, but due to it combining too much shallow low-level information, it does not perform well when dealing with tasks with more complex detection types. The Spp structure uses PPM to obtain multi-scale features for prediction. The advantage is that the detection speed is fast, but the disadvantage is that the resolution of the feature map used in the final prediction is low, and the lack of spatial information leads to inaccurate segmentation of details. Since the Hrnet structure maintains high resolution throughout the prediction process, it is more suitable for tasks that require high spatial information. However, the computational and memory consumption used by maintaining high resolution makes it difficult for detection efficiency to meet practical requirements. The decoder structure of Deeplabv3+ combines the Spp structure and the U-shape structure. Only one low-level feature fusion is performed before the final prediction. It enhances detail accuracy and guarantees detection speed while avoiding excessive low-level information interference. After experimental analysis, the segmentation part of this paper adopts the enhanced deeplabv3+ decoder structure. On the one hand, in order to extract richer high-level features while maintaining high detection speed, we employ an improved DenseASPP. On the other hand, in order to extract more effective low-level features, the low-level feature map of the backbone network adopts the resDWAB module to perform feature enhancement while maintaining the resolution. In summary, the segmentation part first extracts dense multi-scale information from the high-level feature map obtained by the backbone network through the DDWASPP module and then fuses it with the low-level feature map enhanced by the multi-layer residual Atrous Separable Convolution block. Finally, a prediction is made based on the fused feature map.

#### 3.3.1. Densely Connected Depthwise Separable Atrous Spatial Pyramid Pooling Module

In semantic segmentation, the quality of segmentation roughly depends on how much context information can be effectively used. In some scenarios, there is a large variation in object scales, which poses a great challenge to the encoding of high-level features since information at each scale must be encoded. To remedy this problem, Densely connected Atrous Spatial Pyramid Pooling (DenseASPP) was introduced to generate multi-scale features that not only cover a larger scale range but also cover that scale range densely. However, the disadvantage of DenseASPP is obvious; the amount of calculation is too large.

To address this, we plan to transform it with depthwise separable convolutions. There have been many studies demonstrating that a standard convolution can be decomposed into a depthwise convolution and a pointwise convolution without excessive loss of accuracy. In addition, by analyzing DenseASPP, we found that each standard atrous convolution is followed by a point convolution for depthwise information compression. This causes repeated calculations to a certain extent because the standard convolution already includes both pointwise convolution and depthwise convolution.

**Figure 3:**
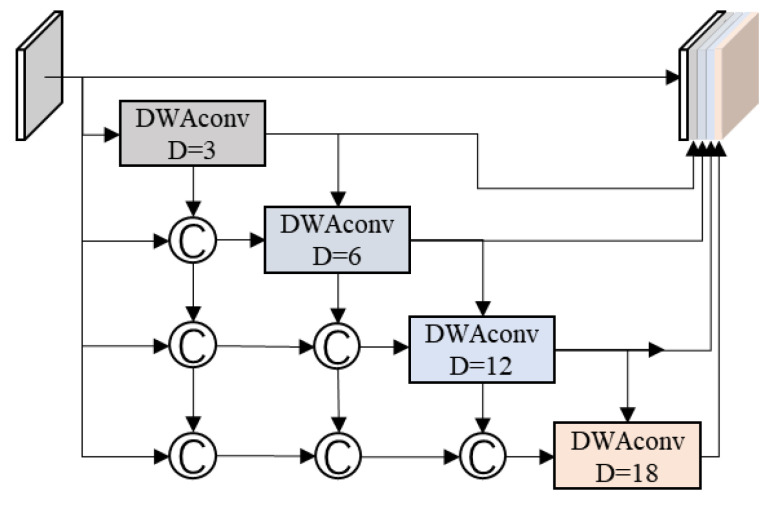
Details of DDWASPP.

Therefore, after replacing the atrous convolution in DenseASPP with the depthwise separable atrous convolution, we cancel the following point convolution. The improved DenseASPP structure is shown in Figure 3.

The DDWASPP module consists of depthwise separable dilated convolutional blocks with different dilated ratios. Each convolutional block is densely connected to obtain sufficient multi-scale information.

Let us analyze the difference in the amount of computation between the DenseASPP and DenseDWASPP modules. If the size of the input feature map is $H_{I}\times W_{I}\times C_{I}$, the convolution kernel size is K, and the size of the output feature map is $\;H_{O}\times W_{O}\times C_{O}$. The calculation amount of standard convolution is S, as shown in Equation (1).

(1) $$S=K^{2}\times C_{I}\times H_{O}\times W_{O}\times C_{O}$$

Depthwise separable convolution is divided into two parts: depthwise and pointwise. The calculation amount of depthwise convolution $S_{DW\;}$ is as shown in Equation (2).

(2) $$S_{DW}=K^{2}\times H_{O}\times W_{O}\times C_{O}$$

Pointwise convolution is actually a standard convolution with a kernel of 1. The amount of calculation is $S_{PW\;}$ as in Equation (3).

(3) $$S_{PW}=C_{I}\times H_{O}\times W_{O}\times C_{O}$$

Total computation of depthwise separable convolution $S_{DSC}$ is equal to the sum of the above two as in Equation (4).

(4) $$S_{DSC}=S_{DW}+S_{PW}$$

The calculation ratio of standard convolution to depthwise separable convolution is:

(5) $$\frac{1}{C_{I}}+\frac{1}{K^{2}}$$

However, since we cancel the point convolution, the calculation ratio becomes $\frac{1}{C_{I}}$, which greatly improves the computational efficiency.

#### 3.3.2. Residually Connected Depthwise Separable Atrous Convolutional Blocks

**Figure 4:**
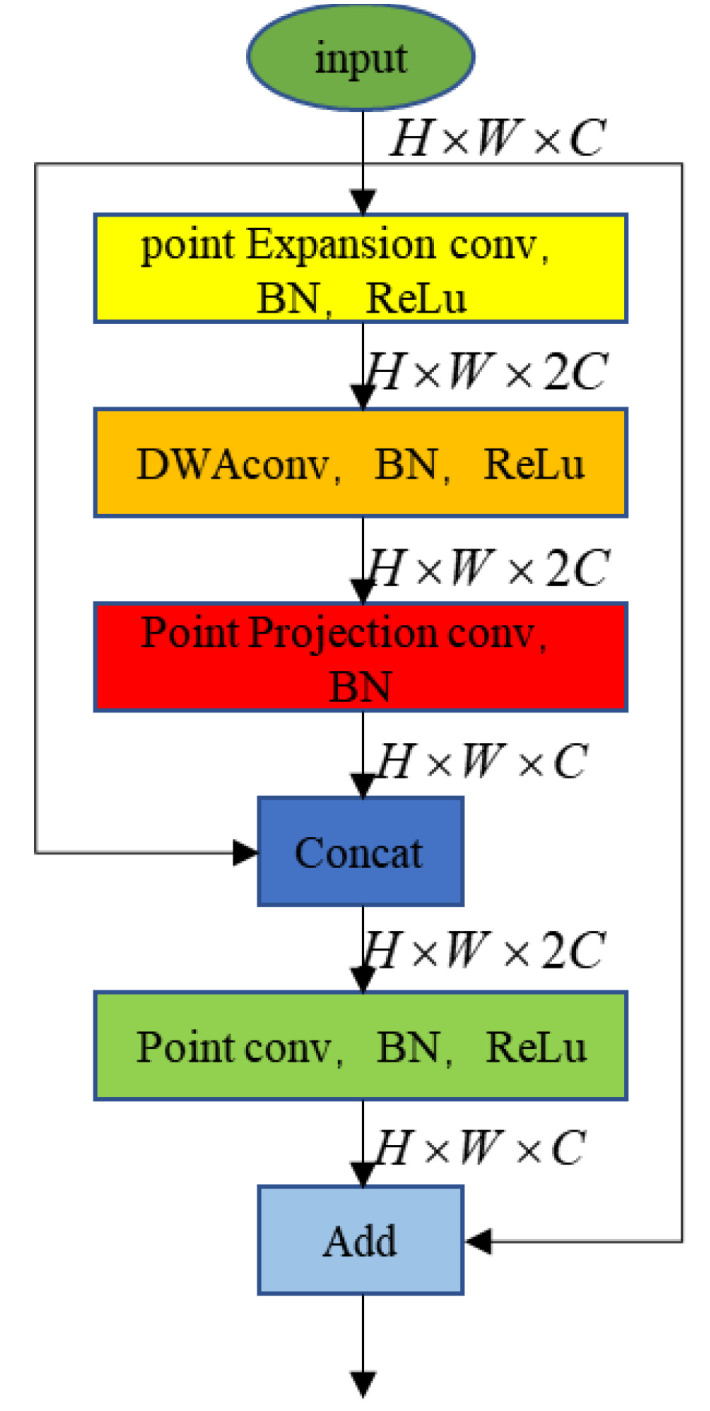
Architecture of the proposed resDWAB.

High-level feature maps have larger receptive fields, richer semantic information, and lower resolution. This results in the loss of spatial detail information. Directly using high-level feature maps for segmentation prediction will result in coarse details. Low-level feature maps have higher resolution and more spatial detail information but lack sufficient semantic information for contextual analysis. Therefore, high-level and low-level feature fusion is required. However, since the backbone network uses fast downsampling, the low-level feature maps are simpler. Only one 6 × 6 convolution and one 3 × 3 convolution are used to downsample the feature map to a quarter of the raw image. It directly causes the spatial features contained in the low-level feature map to be blurred. Not only can it not be used to enhance the effect of the final prediction, but it is also likely to interfere with the prediction results. In order to get better spatial information, it is necessary to increase the depth of the low-level layers. However, the low-level feature map has a higher resolution, so directly increasing the network depth will greatly increase the amount of computation, thereby reducing the running speed of the network. To this end, we design Residually Connected Depthwise Separable Atrous Convolutional Blocks (resDWAB) to improve the spatial information of low-level feature maps at a low computational cost. The structure is shown in Figure 4.

The resDWAB uses an inverted residual model. First, use point convolution to increase the number of channels, then use depth separable atrous convolution for feature extraction, and then use a point convolution to compress the number of channels. Finally, it is concatenated with the features of the previous layer then a point convolution is used for feature fusion. We borrowed the inverted residual structure of MobilenetV2. The depthwise separable convolution is replaced by the depthwise separable atrous convolution, and a feature concatenate path is added to match the atrous convolution to obtain richer multi-scale information. However, an excessive dilation rate will cause a lack of spatial detail information, so we choose to set the dilation rate of the three DWACBs to 0, 1, and 2, respectively.

#### 3.3.3. Deep Supervision

Training becomes difficult as the depth of the network increases because the segmentation decoder has a shorter path to the bottom layer of the encoder. During backpropagation, the underlying parameters of the encoder undergo relatively more drastic changes, which affect the extraction of high-level features of the network. As a result, the performance of the detection decoder that only uses high-level feature maps is significantly degraded. In order to solve the above problems, we adopt a deeply supervised way of training. Deep supervision is used in the PAN layers of the encoder to optimize the high-level layers.

## 4. Loss Function and Evaluation Metrics

In this section, the loss functions and performance evaluation indicators used will be described in detail.

### 4.1. Loss Function

There are two tasks of object detection and semantic segmentation in the multi-task network. Object detection includes three parts: classification, localization, and confidence. The detection part of the localization loss uses *CIOU*. The specific form of *CIOU* positioning loss is shown in Equation (6).

(6) $$CIOU=IOU-\frac{\rho^{2}}{c^{2}}-\alpha v$$

where $\rho^{2}$ represents the Euclidean distance between the center points of the predicted box and the ground-truth box. IOU represents the intersection and union ratio of the two boxes. C is the diagonal length of the smallest rectangle covering both. The positive trade-off parameter of *α* is defined as Equation (7). *V* is used to measure aspect ratio consistency, defined as Equation (8) where $w_{l}$ and $h_{l}$ are the length and width of the true value, and $w_{p}$ and $h_{p}$ are the length and width of the predicted bounding box.

(7) $$\alpha =\frac{v}{1-IOU+v}$$

(8) $$v=\frac{4}{\pi^{2}}{\left(\arctan\frac{w_{l}}{h_{l}}-\arctan\frac{w_{p}}{h_{p}}\right)}^{2}$$

The final positioning loss function is shown in Equation (9).

(9) loss=1−CIOU

The classification, confidence, and semantic segmentation of object detection adopts the cross-entropy function ($loss_{bce}$) to calculate the loss. The batch cross-entropy loss function is shown in Equation (10).

(10) $$L\left(T,P\right)=-\frac{1}{N}\overset{N}{\underset{i=1}{\sum }}\left[T_{i}\cdot \log P_{i}+\left(1-T_{i}\right)\log\left(1-P_{i}\right)\right]$$

where *T* and *P* represent the true value and the predicted value, respectively. *N* represents the number of samples per batch.

The total loss for final object detection is the weighted sum of localization ($loss_{rect}$), classification ($loss_{cls}$) and confidence losses ($loss_{obj}$), defined as Equation (11).

(11) $$loss_{det}=a\times loss_{cls}+b\times loss_{rect}+c\times loss_{obj}$$

where *a*, *b*, and *c* are the weights of the three losses, respectively.

Semantic segmentation loss is directly $loss_{bce}$. The total loss function of the network is shown in the following equation:

(12) $$loss_{all}=\omega loss_{det}+\left(1-\omega \right)loss_{seg}$$

ω stands for the weight of the detection loss, and (1−ω) stands for the weight of the segmentation loss.

### 4.2. Evaluation Method

Semantic segmentation adopts mean intersection-over-union (mIOU) [15] to evaluate the prediction results. Object detection uses mAP@0.5 to evaluate the performance.

## 5. Experimental and Results

In this section, we detail the experimental design and present the experimental results. We demonstrate that our approach achieves a reasonable design and promising results.

### 5.1. Dataset

The NEU-Seg defect dataset is a standardized, high-quality database collected by K. Song and Y. Yan [16] to solve the problem of automatic identification of hot-rolled steel strips. The dataset includes six categories of defects: patches (Pa), inclusion (In), scratches (Sc), crazing (Cr), rolled-in scale (Ri), and pitted surface (Pt).

### 5.2. Training Environment Parameters

The training environment uses ubuntu 18.04, GPU: Tesla-P100 16 GB. CUDA 11.2. python 3.7.13, pytorch 1.12.1. 

Because we have chosen yolov5 as the backbone network, the initialization parameters of the backbone are directly converted from yolov5, which has been well-pre-trained, and the network parameters will be changed more frequently in the multi-task network. Therefore, we use a lower set of super parameters. All networks were trained using the SGD optimizer and cosine annealing method with a learning rate of 10^−4^ and a batch of 32 samples.

### 5.3. Training Strategy

In this section, we mainly explore the impact of training strategies and different weight ratios between multiple tasks on network performance.

#### 5.3.1. Training Method

The training methods of multi-task networks generally include end-to-end training and alternating training. However, in this task, the two decoders are strongly correlated. Deep supervision can make the high-level feature maps of the network have clearer object contour features, which can improve the detection effect to a certain extent. Therefore, we adopt the method of training the segmentation decoder first. First, freeze the detection decoder to train the segmentation decoder and the encoder. Second, freeze the backbone and segmentation decoder to train the detection decoder. Finally, the two tasks are end-to-end trained with a low learning rate for fine-tuning the network. To investigate the effect of training strategy on the result, we conduct a comparative experiment of four training strategies with ω=0.5. The experimental results are shown in Table 1.

As can be seen from Table 1, the best performance is achieved with the training strategy of training segmentation first. The end-to-end training method and the alternating training method achieve similar performance. However, due to the roughness of the detection task relative to the segmentation task, training the detection first does not allow the encoder to learn good features. In the next two stages of training, the network parameters will be greatly optimized, which makes it difficult to achieve good results in the end. Compared with the other three training strategies, training the segmentation task first enables the encoder to learn a relatively finer feature. This has a good guiding effect on the training of the detection decoder later, which makes the final detection perform the best. 

#### 5.3.2. Loss of Weight

Due to the inconsistent loss function of each task in the multi-task network, the difficulty of the task is inconsistent. The loss that results in each task does not stabilize and synchronously converge. The loss of multi-tasking needs to be balanced. To this end, we study the impact of different ω on network performance. The results are shown in Table 2.

As shown in Table 2, semantic segmentation is a relatively easier task to converge in surface defect detection. Even with a weight of only 0.1, it can still obtain a decent result. However, in this case, the segmentation head acts more like an auxiliary loss that can significantly improve the detection performance. The detection task itself is more difficult to converge because it contains three losses. As the detection weight decreases, the performance will gradually decrease.

### 5.4. Ablation Experiment

To verify the effectiveness of our proposed method, we conducted a series of ablation experiments. We removed the detection head on the baseline and used the fused feature maps of FPN as the output of the baseline. Several typical segmentation structures were constructed and tested separately. The experiments used the same dataset partition and training parameter settings. The results are shown in Table 3.

As shown in Table 3, using Aspp and other methods combined with multi-scale information can effectively increase the performance of the network. Our improved DenseAspp module greatly reduces the amount of computation with only a 0.89% performance degradation. At the same time, it can be seen from the last four rows of the table that combining some shallow low-level features not only does not enhance the spatial information but even interferes with the final prediction.

Based on the above experiments, we investigate the impact of low-level information reinforcement modules. Take the setting of the last row of Table 3 as the baseline. Add resDWAB to feature maps whose resolution is one-fourth the size of the original image. We study the effect of the number of resDWAB modules on network performance improvement. The results are shown in Table 4.

It can be seen from Table 4 that with the gradual deepening of the low-level layers, the spatial information of the low-level feature map is gradually clear. At this time, fusion with advanced feature maps can improve the segmentation effect. However, the improvement brought by combining spatial information is limited. When resDWAB was stacked three times, it was almost approaching the upper limit of what the structure could achieve.

### 5.5. Comparative Experiments and Discussion of Results

In this subsection, we compare our method with other methods. The advantages of our proposed method are demonstrated.

#### 5.5.1. Comparative Test

**Figure 5:**
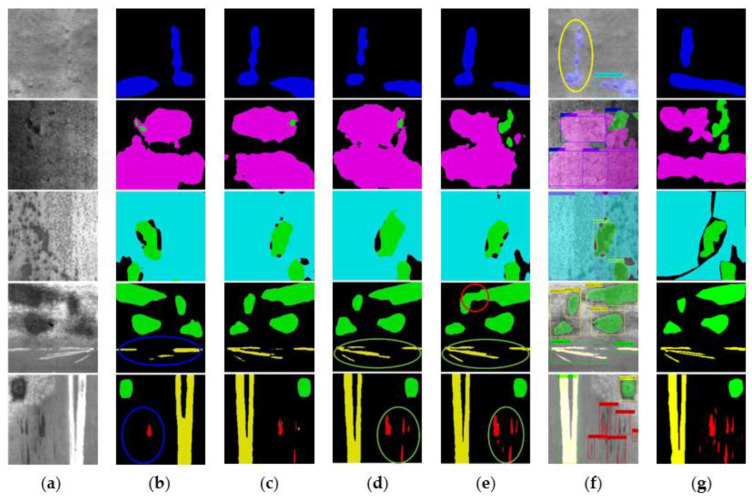
Comparison of segmentation results. (**a**) Original image. (**b**) PSPnet. (**c**) Deeplabv3+. (**d**) Hrnet. (**e**) Unet. (**f**) Ours. (**g**) Ground truth. The colored circles in the figure show false detections.

To verify the effectiveness of the proposed method, we selected some classical algorithms to compare with our method. For the segmentation task, we chose Pspnet, Unet, Hrnet, and Deeplabv3+ networks for comparison. The above four networks are representative networks of four typical structures of semantic segmentation. The detection task selects Faster R-CNN [19], yolov5s. Faster R-CNN is an excellent representative of a two-stage object detection algorithm, and yolov5s is an advanced single-stage network, and it is also the baseline of our work. The defect segmentation visualization is shown in Figure 5.

As can be seen from Figure 5, Unet and Hrnet achieve the best results among segmentation algorithms in the In class and Sc class, which require the most spatial detail information. In the green circle in the figure, Unet and Hrnet achieved the most accurate segmentation. However, Unet performance deteriorated significantly when faced with more complex scenes. As shown in the red circle, Unet cannot distinguish Pa from the background well. Pspnet achieves the worst results on the In and Sc classes because there is not enough spatial information for prediction, as shown by the blue circles in the figure. Furthermore, it can also be seen from the figure that all segmentation algorithms perform worse than our object detection decoder on the class. Unet is still missing many details. However, in the Ri class, our detection decoder is far inferior to the segmentation algorithm. In the yellow circle in the figure, the detection algorithm failed to detect the Ri-type defect, but all the segmentation algorithms segmented this defect relatively accurately. The detailed performance evaluation results are shown in Table 5.

By analyzing the data in Table 5, it can be found that Unet and Hrnet have achieved better results on In and Sc, which require more detailed information. However, because the multi-scale information is not encoded on the high-level feature map, the performance of the Pt class with the largest defect scale is poor. Pspnet achieves the best results in the Pt class. However, it achieves the worst results on In and Sc due to not incorporating low-level features. Deeplabv3+ takes into account both high-level and low-level features and achieves the best balance between speed and performance among several classical algorithms. Our model strengthens both high-level and low-level features on the one hand and benefits from the training advantages of multi-task networks on the other hand. Finally, our model achieved the best performance.

**Figure 6:**
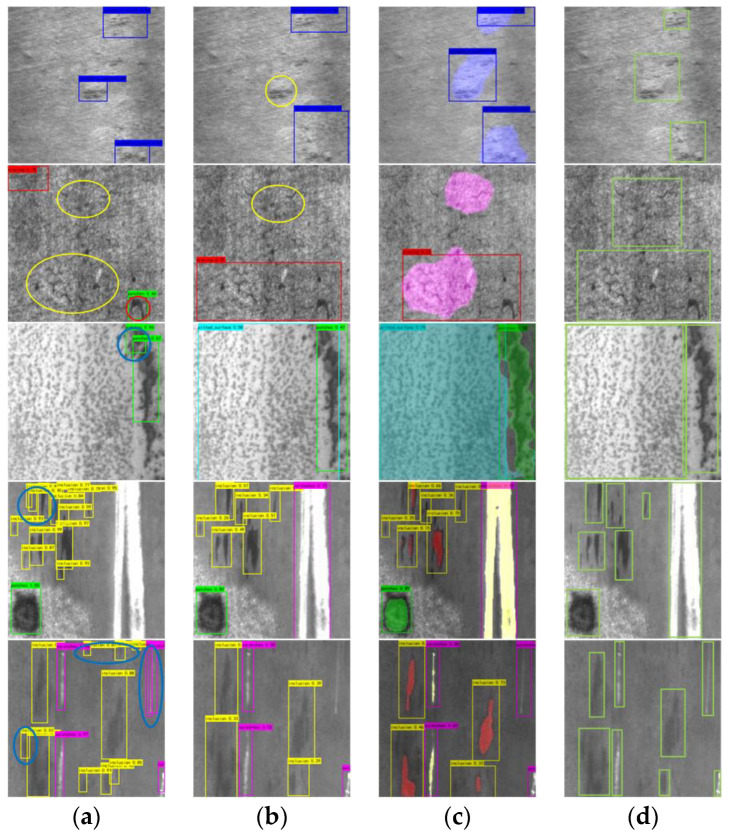
Comparison of detection results. (**a**) Faster-RCNN. (**b**) yolov5s. (**c**) Ours. (**d**) Ground truth. The colored circles in the figure show false detections.

The comparison result of the object detection algorithm is shown in Figure 6. 

As can be seen from Figure 6, Faster-RCNN has the worst detection performance. Because the two-stage algorithm cannot effectively utilize the contextual information, this directly leads to the model misclassifying the target. In the red circle in the figure, Faster-RCNN mistakenly identified a part of the Cr class as the Pa class. Furthermore, the fixed-size detection boxes of the two-stage model are difficult to adapt to the diversity of defect scales, which leads to many single defects being identified by multiple detection boxes, and the defect range cannot be correctly framed, as shown by the blue circle in the figure. The single-stage detection model does not have the same problems as the two-stage model due to the full use of context information. However, neither the single-stage nor the two-stage model can handle the Ri and Cr classes with blurred boundaries well, as shown by the yellow circle in the figure. The single-stage detection model does not have the same problems as the two-stage model due to the full use of context information. However, neither the single-stage nor the two-stage model can handle the Ri and Cr classes with blurred boundaries well, as shown by the yellow circle in the figure. The performance metrics of the different algorithms are depicted in Table 6.

As shown in Table 6, we compare our model with yolov5s, Faster-RCNN, DIN of Hao et al. [35], and DDN of He et al. [64] Faster-RCNN achieves the worst detection performance due to its fixed detection window and underutilization of contextual information. Hao has made many improvements on the basis of Faster-RCNN and achieved a large performance improvement. The model has achieved the best results, but the improvement is limited, and the detection speed is too low to be practical. Our model benefits from the advantages of multi-task networks; the mAP metric is 0.69% higher than the original yolov5s. A detailed analysis of the indicators of specific defect categories shows that the Pa indicator dropped by 4.33%. It should be because the segmentation effect of the Pa class is not as good as the detection effect, which drags down the performance of the detection to a certain extent. The Cr class is also reduced because the segmentation effect is not good enough. Pt and Ri obtain higher enhancements from the segmentation-optimized feature maps, increasing by 3.23% and 6.14%, respectively.

In addition, it can be seen from Figure 5 and Figure 6 and Table 5 and Table 6 that object detection and semantic segmentation each have their own advantages and disadvantages in the task of defect detection. Object detection has better results on tasks with obvious target characteristics such as In, Pa, and Sc. Semantic segmentation works better on objects with more obvious texture features such as Cr, Ri, and Pt. Through multi-task learning, the advantages of the two can be efficiently combined at a lower computational cost. To sum up, our proposed method has obvious advantages in detection accuracy and practicality.

#### 5.5.2. Failure Case Analysis

**Figure 7:**
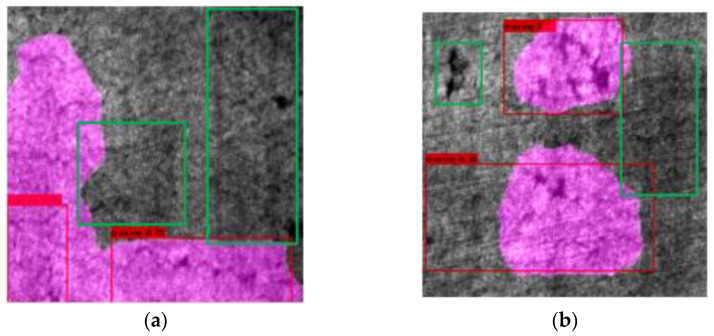
Failure analysis. (**a**,**b**) show failed predictions. The green boxes in the figure are undetected defects.

The failure analysis result is shown in Figure 7. The green boxes in the figure are undetected defects.

From Figure 7, although our model achieves good results in general, there are still some defects that are not accurately detected. We will try to explore the reasons for the unsatisfactory test results. The error cases in this model mainly occur in the Cr class. Neither segmentation nor detection can detect such defects well. We think there are two main reasons. (1) The background difference of Cr-type defects is very insignificant. It is difficult for our method to separate it from the background. (2) The boundaries of adjacent defects are not obvious, which makes object detection prone to wrongly delineating the defect range. The boundary is not clear. Not even humans can pinpoint its extent.

## 6. Conclusions

In this work, we propose a multi-task network for steel surface defect inspection. The network combines the advantages of object detection and semantic segmentation at the same time. Six major defects can be detected in real-time. The model achieved mIOU: 79.37%, mAP@0.5: 78.38%, and the detection speed reached 85.6 FPS on a single GPU. In the framework, multi-level features from defect images are extracted by the encoder. The object detection task adopts the Path Aggregation Network structure to convey spatial information to strengthen the prediction results. The semantic segmentation task adopts the Densely connected Depthwise Separable Atrous Spatial Pyramid Pooling module to extract dense multi-scale information and uses Residually Connected Depthwise Separable Atrous Convolutional Blocks to reinforce low-level features. Deep supervision is used in the framework to improve optimization and maintain the training advantage of multi-task networks. The training strategy is improved to improve the overall performance of the model further. Experiments show that our proposed method has obvious advantages over previous work. However, although our model achieves good results, there are still some defects that are not well detected, as shown in Figure 7. In future research, we will further study more reasonable detection methods so that the detection performance of all types of defects can meet the actual detection needs well.

## Figures and Tables

**Table 1:** Metrics of Different Training Strategies.

Method	mIOU	mAP@0.5
end-to-end training	79.21	77.33
rotation training	78.85	77.46
first-train detection	78.79	77.21
train the segmentation first	79.51	77.52

**Table 2:** Metrics of The Proposed Network with Different Weights.

ω	mIOU	mAP
1	2.3	77.69
0.9	73.25	79.62
0.8	75.53	79.12
0.7	78.91	78.47
0.6	79.37	78.38
0.5	79.51	77.52
0.4	79.18	74.37
0.2	78.90	67.83
0.1	78.81	63.62
0	78.75	0.2

**Table 3:** Detailed Performance of Our Method with Different Settings.

Method	mIOU
Baseline	76.51
Baseline + Aspp	77.13
Baseline + DenseAspp	78.57
Baseline + DWAspp	77.68
Baseline + U-shape decoder	73.82
Baseline + v3plus decoder	74.63
Baseline + Aspp + v3plus decoder	75.37
Baseline + DWAspp + v3plus decoder	75.84

**Table 4:** Detailed Performance of Our Model with Different resDWAB Numbers.

Method	mIOU
Baseline + resDWAB × 1	77.52
Baseline + resDWAB × 2	78.34
Baseline + resDWAB × 3	78.98
Baseline + resDWAB × 4	79.37
Baseline + resDWAB × 5	79.42

**Table 5:** Quantitative Comparisons of Different Segmentation Methods.

Method	mIOU	Ba	In	Pa	Sc	Cr	Ri	Pt	FPS
Pspnet [41]	75.76	96.03	64.48	84.07	73.22	48.11	74.59	89.83	26.6
Unet [40]	74.62	95.75	67.21	83.36	78.51	45.32	71.73	80.43	60.1
Hrnet [42]	75.86	96.15	69.57	84.78	79.01	49.55	71.76	80.18	24.9
Deeplabv3+ [37]	77.90	96.08	67.69	83.72	77.74	58.43	75.03	86.64	65.8
Ours	79.37	96.17	69.91	85.23	78.76	61.47	74.84	89.22	85.6

**Table 6:** Quantitative Comparisons of Different Detection Methods.

Method	mAP@0.5	In	Pa	Sc	Cr	Ri	Pt	FPS
Yolov5s	77.69	81.15	96.26	87.91	52.34	65.66	82.83	160.5
Faster-RCNN [64]	66.12	72.41	77.77	84.43	31.46	58.91	71.48	10.2
He [64]	82.31	84.7	90.7	90.1	62.4	76.3	89.7	6.25
Hao [35]	80.38	85.55	93.01	88.26	61.39	63.75	90.33	43.5
Ours	78.38	81.58	91.93	88.75	50.17	71.80	86.06	85.6

## Data Availability

All data, models, and code generated or used during the study appear in the submitted article.
